# Advances in Occupational Health in Guatemala

**DOI:** 10.29024/aogh.2319

**Published:** 2018-08-31

**Authors:** M.E. González Alvarez, M.C. Guzmán-Quilo

**Affiliations:** 1 Center of Occupational and Environmental Health SOA SALTRA, CA; 2 Department of Toxicology, School of Pharmaceutical Chemistry, Faculty of Chemical Sciences and Pharmacy, University of San Carlos of Guatemala, GT

## Abstract

Occupational health in Guatemala has come a long way. In 1958, the first Regulation of Occupational Health by the Guatemalan Social Security Institute was published. There wasn’t another Directive in the country regarding this issue until the year 2000, when the National Council for Occupational Safety and Health was created. In 2014, it published the Governmental Agreement 229-2014 Occupational Health and Safety Regulations, which came into force on September 8th, 2015. Nowadays there are other institutions that care about this topic. Some of these institutions promote occupational health training through courses, workshops, seminars, etc., but there is not a formal education program yet. There are some other institutions, such as the National Institute of Statistics, which generates information concerning employment, unemployment, characteristics, composition, structure, and functioning of the labor market through surveys. And finally, there are other institutions like the Health, Labor and Environment Program of Central America SALTRA, which promotes investigation in this topic, generates information as well as endorses training regarding occupational safety as an important issue in the country.

Guatemala (Figure 1) is a country located in Central America. It has a territorial extension of 108,889 km^2^ and an approximate population of sixteen million habitants. It has a human development index of 0.640, placing it in 125th place for human average development, only above Honduras, out of all the countries of the Central American Isthmus [1,2]. As a country, there is still a long way to go and the issue of occupational health is no exception.

**Figure 1:**
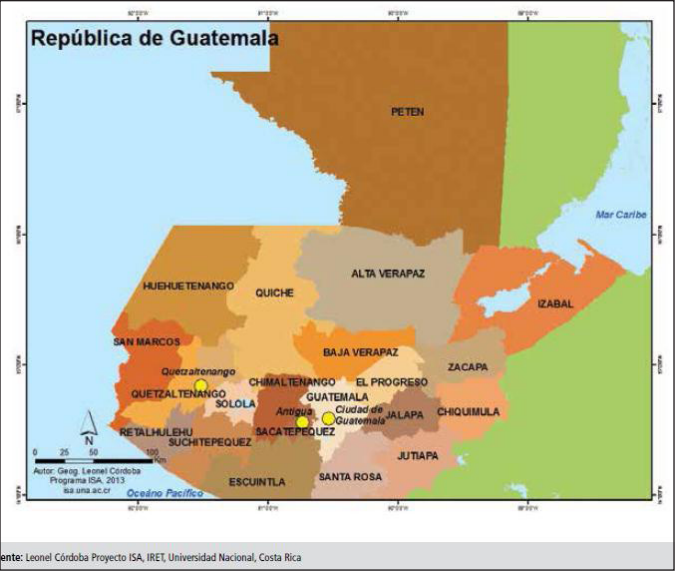
Map of the Republic of Guatemala [7]. Source: Leonel Córdoba Proyecto ISA, IRET, Universidad Nacional, Costa Rica [7].

Until a few years ago, occupational health in Guatemala was a subject that nobody talked about, and there was very little knowledge on it. In 1958 the General Regulation on Hygiene and Safety at the Workplace came into force. From that time until the year 2014, when the Governmental Agreement 229-2014 Occupational Health and Safety Regulations was published, there were no other regulations regarding the health of workers. Of 189 ILO Conventions, Guatemala had ratified 73 (38.6%) by the year 2015. Of this 189 ILO Conventions 13 were relevant to occupational health and safety and Guatemala only had ratified 5 (38.5%) [7,8].

The National Council for Occupational Safety and Health (Consejo Nacional de Salud y Seguridad Ocupacional – CONASSO) was created in 2000, according to Ministerial Agreement 314-2000, based on ILO Convention No. 187, which has not been ratified by Guatemala. According to Hernández, M. et al. (2013), CONASSO’s mission is to coordinate, advise and design proposals to promote national policies, as well as formulate strategies and promote actions in occupational safety and health [7]. This council is tripartite, comprising the government sector, the employer sector, and the labor sector. Some of the achievements of CONASSO are: The Governmental Agreement 229-2014 Occupational Health and Safety Regulations that came into force on September 8th, 2015, and its reforms found in Governmental Agreement 33-2016 that came into force on February 13th, 2016. Both governmental agreements were created due to the need in the country to update the general conditions of hygiene and safety practices in the workplace for both the employer as well as employees. Due to these changes, the State can ensure the health and safety of the working population of the country. The second agreement was created to clarify dispositions in Governmental Agreement 229-2014 that could be questioned or misinterpreted. Another achievement of CONASSO was the 191-2010 Decree. This decree obligates the employer to report work accidents to the Ministry of Labor and Social Security (Ministerio de Trabajo y Previsión Social – MINTRAB) [3,4,6,7].

Despite all of these efforts, there are still many shortcomings that do not meet the demands of occupational health and safety in the country. Although there are some institutions, organizations, and technical and scientific associations that are interested in this issue and promote occupational health training through courses, workshops, seminars, etc., there is not a formal education program and the country does not have enough trained staff. For example, by 2015 the ratio of labor or hygiene and safety inspectors was 305 per 5,988,175 economically active population occupied. The ratio of professional graduates or postgraduates in SSHO by the year 2015 was 0.0000005 (3 professional graduates per 5,988,175 economically active population occupied) and the three of them graduated abroad. There were 55 physicians working in occupational health per 1,837,449 people of the occupied population of the formal sector of economy in the country and two nurses working in occupational health during the year 2015. In addition, it should be mentioned that another factor influencing the statistics above is the lack of resources needed to cover the demand that occupational health entails for every worker [7,8].

In 2016, the Guatemalan Social Security Institute (Instituto Guatemalteco de Seguridad Social, IGSS) represented only 22.9% of the economically active population. This means that almost 70% of the economically active population did not have social security because they were part of the informal sector of the economy. They are therefore forced to use national hospitals, which have been crippled by the economic crisis that manifests in the country; the hospitals lack the necessary tools to deal with diseases and accidents in the workplace. It is also important to mention that Guatemala does not yet have a national list of occupational diseases, which makes it very difficult to determine and prevent many of these diseases. The fact that a large part of the population belongs to the informal sector of the economy affects not only whether or not people have access to social security, but it also affects the economic income for families. They often can’t meet basic needs, such as food, education, housing, and health, let alone cover expenses related to occupational health. Child labor is another fatal consequence of the economic problems faced by families in the country. By 2015, 9.5% of children and adolescents aged seven to 17 will have engaged in the workforce and will not have the opportunity for an education. At the national level, by the year 2016, 6.3% of children between seven and 14 years of age will have already engaged in some type of economic activity [5,8].

Other entities, such as the National Institute of Statistics (Instituto Nacional de Estadística, INE), have also made efforts to obtain information on occupational health, incorporating some questions related to it on the National Employment and Income Survey (Encuesta Nacional de Empleo e Ingresos – ENEI) from the first semester of 2013 up to today. In 2015, Mr. Ruben Narciso, INE’s manager, indicated that the ENEI produces information that makes it possible to know the behavior and evolution of employment, unemployment, characteristics, composition, structure, and functioning of the labor market [9]. One of the questions included is related to risk factors at work. For the second half of 2014, extreme temperature was shown to be the most frequent risk factor (36%), with humidity being the second most frequent risk factor (31%). The least frequent risk factor (8%) is illumination. Other risk factors to consider are: smoke and dust, noise, toxic substances, and vibrations. Twenty perent of the workers surveyed indicated they needed to be provided with personal protective equipment in order to carry out their work; of these workers, 24% stated that they did not have adequate protective equipment during working hours [9].

The Health, Labor and Environment Program in Central America (Salud, Trabajo y Ambiente en América Central, SALTRA) is a program that was created in 2003, with the objective of developing national and regional capacities in Central America for the prevention of occupational hazards, with public health and sustainable production as a necessary condition to reduce and prevent poverty in the region [10]. The regional coordination of this program is in charge of the National University of Costa Rica. Each country member of the Central American isthmus has its own national coordinating institutions of the SALTRA program in the state universities; in Guatemala, the headquarters is the Department of Toxicology, of the Faculty of Chemical Sciences and Pharmacy of the University of San Carlos of Guatemala (staff of the Department of Toxicology in Figure 2). This program, in its first phase, from 2003 to 2009 was funded by the Swedish Cooperation Agency for Human Development. The second phase began in 2011, where the European Union was responsible for financing this project and sought to retake and expand towards environmental health the achievements that were obtained in occupational health during phase one. This second phase ended in 2015. Currently, the Center of Occupational and Environmental Health of Guatemala works thanks to the staff that works in the Department of Toxicology with the funds generated by this department [10].

**Figure 2:**
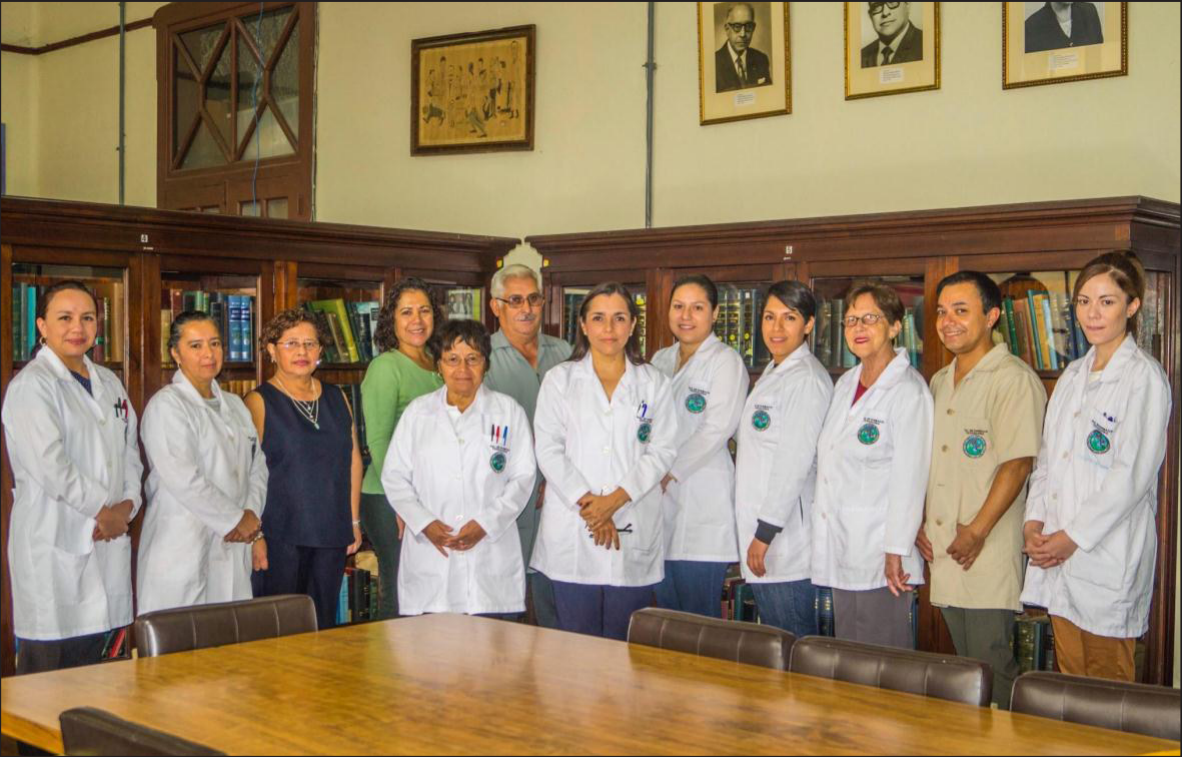
Staff of the Department of Toxicology, of the Faculty of Chemical Sciences and Pharmacy of the University of San Carlos of Guatemala. Source: Sandoval, O., 2017.

The objectives of the SALTRA Center in Guatemala lie in helping to prevent risks and diseases in the labor sector in such a way that it becomes a technical-scientific entity that can support initiatives to improve the health of the working population [10]. It is also important to mention that, since the first phase, this center has a commitment to guide preventive policies that help improve occupational health by monitoring indicators that evaluate and monitor the trends for health risks and their effects on the working population of the region [10]. Some of the publications by the SALTRA program include: Occupational Health Profile in Guatemala, 2013, Estimated number of workers exposed to selected carcinogens and pesticides in Guatemala, 2015, and the Occupational Health and Environmental Indicators Profile Guatemala, 2015 [10]. On Figure 3, some members of the SALTRA team and the Department of Toxicology working at the SALTRA Center, Department of Toxicology of the Faculty of Chemical Sciences and Pharmacy of the University of San Carlos of Guatemala.

**Figure 3:**
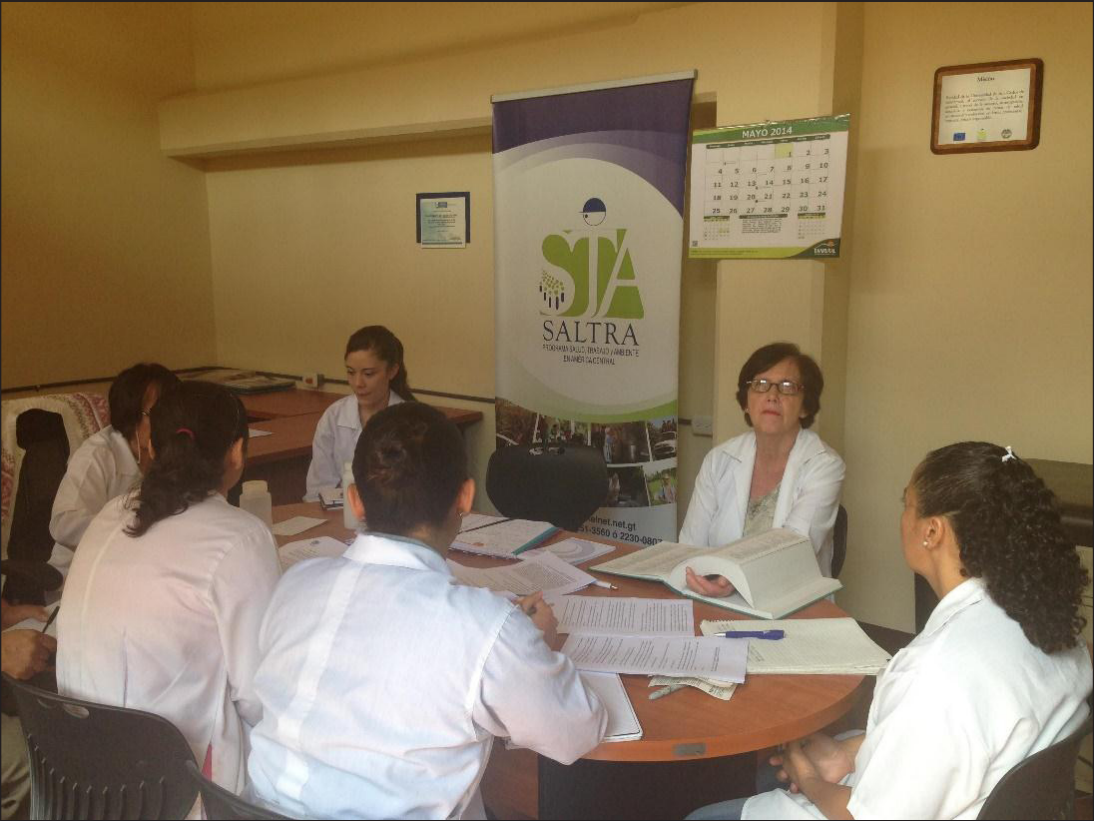
SALTRA Center, Department of Toxicology, of the Faculty of Chemical Sciences and Pharmacy of the University of San Carlos of Guatemala. Source: Guzman-Quilo, C., 2016.

Guatemala has recently started moving from having no advocacy for workplace rights to getting involved with several institutions, some of them mentioned in this article. Although the country is still working to improve occupational health, a lot remains to be done, and the rights of employees are only addressed in their most basic form. However, it is a start that awakens social awareness on the issue in Guatemala. Entities are working every day to make the workforce safer and better, but a lot must still be accomplished, especially in terms of political will.
